# The Global Success of Mycobacterium tuberculosis Modern Beijing Family Is Driven by a Few Recently Emerged Strains

**DOI:** 10.1128/spectrum.03339-22

**Published:** 2023-06-05

**Authors:** Chendi Zhu, Tingting Yang, Jinfeng Yin, Hui Jiang, Howard E. Takiff, Qian Gao, Qingyun Liu, Weimin Li

**Affiliations:** a Beijing Chest Hospital, Capital Medical University, Beijing, China; b Beijing Tuberculosis and Thoracic Tumor Research Institute, Beijing, China; c Hangzhou Red Cross Hospital, Zhejiang, China; d Instituto Venezolano de Investigaciones Científicas, Instituto Venezolano de Investigaciones Científicas, Caracas, Venezuela; e Key Laboratory of Medical Molecular Virology (Ministry of Education/National Health Commission/Chinese Academy of Medical Sciences), School of Basic Medical Sciences, Shanghai Medical College, Shanghai Institute of Infectious Disease and Biosecurity, Fudan University, Shanghai, China; f Department of Immunology and Infectious Diseases, Harvard T.H. Chan School of Public Health, Boston, Massachusetts, USA; CNRS – University of Toulouse

**Keywords:** *Mycobacterium tuberculosis*, Beijing family, modern Beijing sublineage, evolution, genomic epidemiology

## Abstract

Strains of the Mycobacterium tuberculosis complex (MTBC) Beijing family aroused concern because they were often found in clusters and appeared to be exceptionally transmissible. However, it was later found that strains of the Beijing family were heterogeneous, and the transmission advantage was restricted to sublineage L2.3 or modern Beijing. In this study, we analyzed the previously published genome sequences of 7,896 L2.3 strains from 51 different countries. Using BEAST software to approximate the temporal emergence of L2.3, our calculations suggest that L2.3 initially emerged in northern East Asia during the early 15th century and subsequently diverged into six phylogenetic clades, identified as L2.3.1 through L2.3.6. Using terminal branch length and genomic clustering as proxies for transmissibility, we found that the six clades displayed distinct population dynamics, with the three recently emerged clades (L2.3.4 to L2.3.6) exhibiting significantly higher transmissibility than the older three clades (L2.3.1 to L2.3.3). Of the Beijing family strains isolated outside East Asia, 83.1% belonged to the clades L2.3.4 to L2.3.6, which were also associated with more cross-border transmission. This work reveals the heterogeneity in sublineage L2.3 and demonstrates that the global success of Beijing family strains is driven by the three recently emerged L2.3 clades.

**IMPORTANCE** The recent population dynamics of the global tuberculosis epidemic are heavily shaped by Mycobacterium tuberculosis complex (MTBC) strains with enhanced transmissibility. The infamous Beijing family strain stands out because it has rapidly spread throughout the world. Identifying the strains responsible for the global expansion and tracing their evolution should help to understand the nature of high transmissibility and develop effective strategies to control transmission. In this study, we found that the L2.3 sublineage diversified into six phylogenetic clades (L2.3.1 to L2.3.6) with various transmission characteristics. Clades L2.3.4 to L2.3.6 exhibited significantly higher transmissibility than clades L2.3.1 to L2.3.3, which helps explain why more than 80% of Beijing family strains collected outside East Asia belong to these three clades. We conclude that the global success of L2.3 was not caused by the entire L2.3 sublineage but rather was due to the rapid expansion of L2.3.4 to L2.3.6. Tracking the transmission of L2.3.4 to L2.3.6 strains can help to formulate targeted TB prevention and control.

## INTRODUCTION

Tuberculosis (TB) is the most lethal infectious disease, causing over a million deaths a year (1). As it coevolved with the human host, the Mycobacterium tuberculosis complex (MTBC)—the causative agent of tuberculosis—separated into the nine currently recognized phylogenetic lineages (L1 to L9) (2–4). While some lineages are predominantly associated with particular geographic regions, the global distribution of lineage 2 is believed to reflect its greater transmission capacity (5–8). Lineage 2 (L2), commonly referred to as the Beijing family, was first identified by van Soolingen et al. in 1995 (9) and has been responsible for many large tuberculosis outbreaks in diverse geographic regions (10–12). Early molecular typing techniques, such as spoligotyping and restriction fragment length polymorphism (RFLP), found that Beijing family strains shared very similar or identical genomic footprints, and therefore the Beijing family was thought to comprise a recently expanded clonal population (13–15). Subsequently, whole-genome sequencing revealed substantial genetic diversity among MTBC Beijing family strains and showed that over the past 1,800 years, the family has evolved into several distinct sublineages (5, 8, 16).

The common ancestor of L2, the MTBC Beijing family, likely originated in South East Asia and then diverged into three major sublineages: L2.1 (proto Beijing), L2.2 (ancient Beijing), and L2.3 (modern Beijing) (5, 17). Among these, the L2.1 strains are only occasionally isolated and are prevalent only in southern East Asia (18). L2.2 strains are widely distributed in East Asia but less prevalent in regions outside Asia (8). L2.3 strains, however, have a worldwide distribution and account for greater than 70% of Beijing family strains isolated globally (5, 8, 19). We previously determined that L2.3 strains emerged from the L2.2 strains about 500 years ago (8) and are more readily transmitted than L2.2 strains in regions where both sublineages are present (8). In addition, L2.3 strains appeared to be more virulent than L2.2 strains in animal infection models (20). While it was originally thought that increased transmissibility was a characteristic of the entire Beijing family, there is now a consensus that the transmission advantage is a characteristic only of strains belonging to sublineage L2.3 (8, 21).

Over the past ~500 years, sublineage L2.3 has diverged into several phylogenetic clades separated by tens to hundreds of single nucleotide polymorphisms (SNPs) (16, 19, 22, 23). In this study, we sought to determine whether the global expansion and transmission advantage of the Beijing family strains were attributable to the entire sublineage L2.3 or only a subset of the L2.3 clades. By analyzing the genomes of 7,896 L2.3 strains from 51 countries, we show that the global spread of the Beijing family was largely attributable to just three recently emerged clades within sublineage L2.3.

## RESULTS

### Northern East Asia origin of sublineage L2.3.

**FIG 1:**
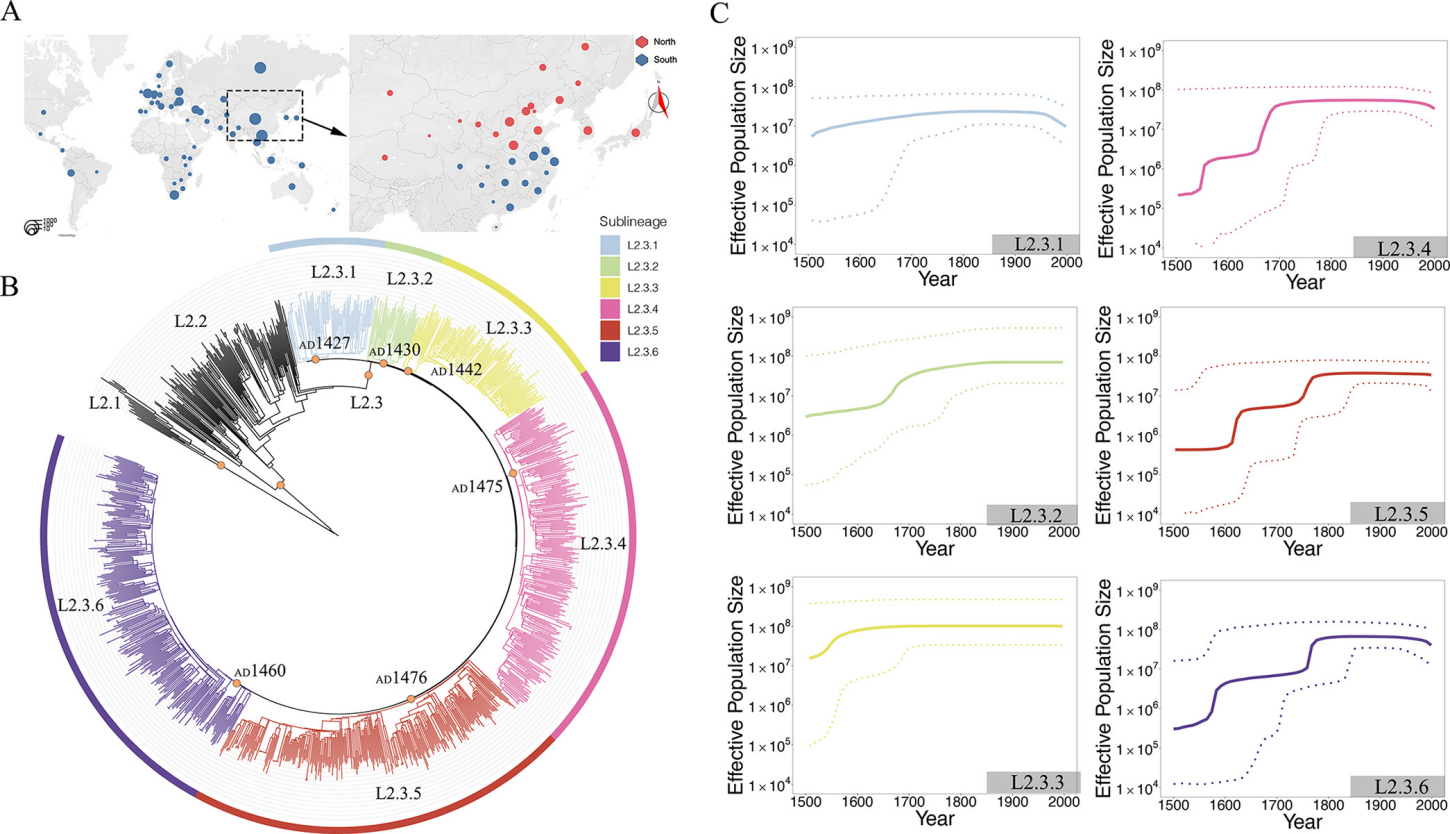
Phylogenetic structure and inferred population dynamics of L2.3 clades. (A) The geographic distribution of MTBC L2.3 strains included in this study (left) and the geographical division of strains from North and South East Asia (right). (B) A maximum-likelihood phylogenetic tree of L2 strains used from East Asia, among which L2.3 is defined as six clades L2.3.1 to L2.3.6. (C) Bayesian skyline plots for the dynamics of effective population size (*N_e_*) of six L2.3 clades.

To characterize the genetic diversity and population structure of the L2.3 sublineage, we curated the whole-genome sequences of 7,896 L2.3 isolates that had been published in more than 62 studies that sampled strains from 51 countries (Methods). The distribution of these L2.3 strains by continent was as follows: Asia, 3,805; Europe, 2,945; Africa, 652; Oceania, 236; and North/South America, 258 (Fig. 1A; Table S1). We also compared the relative prevalence of the three L2 sublineages (L2.1, L2.2, and L2.3) and found that 94.1% of L2 strains isolated outside East Asia belong to L2.3, while the L2.1 and L2.2 sublineages were largely restricted to East Asia and accounted for only 5.9% of the L2 strains isolated outside this region (Table S2). This is consistent with a previous estimation that most of the world’s L2 strains belong to the L2.3 sublineage (16, 23, 24).

To delineate the evolutionary origin of L2.3 strains, we reconstructed a phylogenetic tree using the 7,896 L2.3 strains (Fig. S1A). The tree showed that the early diverged branches were exclusively sampled from East Asia, while the strains from other continents were mostly found in recently diverged or terminal branches (Fig. S1A). This pattern is consistent with previous work suggesting that L2.3 strains originated in East Asia (5, 8, 25). When we separated the East Asian L2.3 strains isolated in southern East Asia from those from northern East Asia (Fig. 1A), a geographic origin reconstruction analysis with RASP (26) showed that L2.3 strains originated in northern East Asia (posterior probability: 91.0%). In addition, all of the strains on the early branching nodes of the phylogenetic tree were isolated in northern East Asia (Fig. S1B), suggesting that the early expansion of L2.3 strains occurred in this geographic region. We estimated the time to the most recent common ancestor (MRCA) of the L2.3 sublineage as around 591 years ago (95% CI, 292 to 808) (Fig. 1B), implying that this sublineage emerged approximately 1,200 years after the ancestor of lineage 2 diverged from the other MTBC lineages (8).

### Distinct population dynamics of the six L2.3 clades.

To characterize the genetic diversification and population dynamics of the different L2.3 clades, we reconstructed a phylogenetic tree with just the 1,246 L2.3 strains isolated in East Asia. This phylogenic reconstruction identified six clades (L2.3.1 to L2.3.6) with bootstrap values of >90% for each node (Fig. 1B), but the relative prevalence of the six clades varied substantially. Clades L2.3.4 to L2.3.6 accounted for 24.8%, 25.3%, and 28.1%, respectively, of the total 1,246 strains, while L2.3.1 to L2.3.3 clades accounted for only 7.5%, 3.8%, and 10.5% of the strains, respectively (*P* < 0.001, chi-square test). While early emerging clades of MTBC can have larger population sizes (27), this cannot explain the differences in the L2.3 clade prevalence because the six clades emerged over relatively similar time scales, and clades L2.3.4 to L2.3.6 diverged slightly later than clades L2.3.1 to L2.3.3 (Fig. 1B).

We next used Bayesian skyline plots to trace the historical population dynamics of the six L2.3 clades, indicated as effective population size (*N_e_*). While the *N_e_* of clades L2.3.1 to L2.3.3 remained flat over time, the populations of L2.3.4 to L2.3.6 appeared to have undergone multiple periods of major expansion (Fig. 1C). We then calculated the expansion rate of the effective population (*R_Ne_*) over 10-year intervals for each clade and found that clades L2.3.4 to L2.3.6 had significantly higher *R_Ne_* than clades L2.3.1 to L2.3.3 (*P* = 0.002, chi-square test) (Fig. 1C; Table 1).

**TABLE 1:** L2.3 Population expansion rates of the six subclades

Sublineage	Time of appeared (AD)	Growth interval (yr)	Growth rate by interval *R_Ne_* (%)
L2.3.1	1427	312	0.47
L2.3.2	1430	380	0.83
L2.3.3	1442	326	0.68
L2.3.4	1475	282	1.84
L2.3.5	1479	229	1.77
L2.3.6	1483	295	1.72

### L2.3 clades differed in the rates of ongoing transmission.

**FIG 2:**
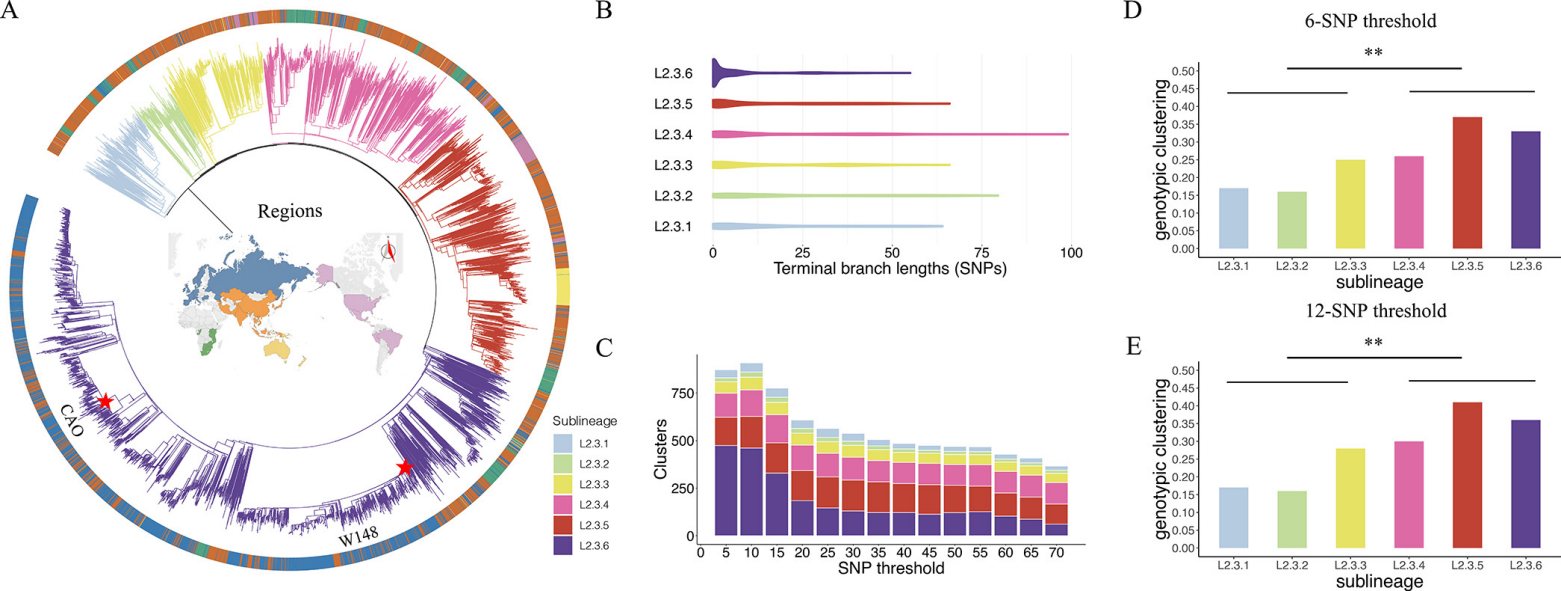
L2.3 clades differed in the rates of ongoing transmission. (A) A maximum-likelihood phylogenetic tree of 7,891 L2.3 strains with geographic information annotated (outer ring). (B) Comparison of terminal branch length (TBL) of the six L2.3 clades. (C) Comparison of cluster numbers between the six L2.3 clades under different single nucleotide polymorphism (SNP) thresholds. (D, E) Comparison of genomic cluster rates between the six L2.3 clades in a population-based data set from Shanghai, China, using 6-SNP and 12-SNP thresholds, respectively. **, *P* < 0.001.

To better characterize the differences in transmission dynamics, we calculated the terminal branch lengths (TBLs) on the phylogenetic tree for each L2.3 clade (Fig. 2A). TBL has been used as an indicator of the expansion capability of bacterial populations, with smaller TBLs representing faster expansion (6). Here, TBL was calculated as the minimum SNP distance between each strain and the common ancestor shared with its nearest neighbor (Fig. 2B). MTBC strains from previously annotated outbreaks were excluded from this analysis. We found that clades L2.3.4 to L2.3.6 had significantly shorter TBLs than clades L2.3.1 to L2.3.3 (Fig. 2B) (*P* < 0.001, Wilcoxon rank-sum test), suggesting that the MTBC strains in clades L2.3.4 to L2.3.6 were likely the result of recent transmission. We repeated the analysis by recalculating the TBLs of isolates circulating in countries where all six clades of the L2.3 were present. Once again, we found that the TBLs of L2.3.4 to L2.3.6 were significantly shorter (Fig. S2). We then estimated the transmission dynamics by counting the number of genomic clusters at different SNP thresholds and found that the majority of genomic clusters in L2.3 were composed of strains belonging to the L2.3.4 to L2.3.6 clades (Fig. 2C). For example, using a 5-SNP threshold, there were 872 genomic clusters, 749 of which (85.9%) belonged to L2.3.4 to L2.3.6 clades (Fig. 2C).

To confirm that L2.3.4 to L2.3.6 clades had higher transmission rates than the L2.3.1 to L2.3.3 clades, we analyzed data from three studies in populations in which all of the clades were circulating: two from China and one from Vietnam. In all three cohorts, the L2.3.4 to L2.3.6 clades had higher clustering rates (6-SNP, *P* = 0.003; 12-SNP, *P* = 0.002; chi-square test), indicating more ongoing transmission (Fig. 2D and E; Fig. S3). Interestingly, when we tested each clade individually, we found that the difference in clustering rates between L2.3.3 and the L2.3.4 to L2.3.6 clades was not significant (*P* = 0.832). Clade L2.3.3 appears to have an intermediate transmission capability, and therefore a binary division into highly transmissible or not highly transmissible clades may not accurately describe the heterogeneity of all L2.3 clades. We then compared the clustering rate of clades L2.3.4 to L2.3.6 with the clustering rate of other sublineages that were also present in the population-based data sets: L2.2, L4.2, L4.4, and L4.5 (Table S3). The clustering rates of these other sublineages were very similar to the rates of clades L2.3.1 to L2.3.2 (*P* = 0.343; chi-square test) and clearly different from the higher rates of clades L2.3.4 to L2.3.6 (*P* = 0.012; chi-square test), again confirming the exceptional transmissibility of the three L2.3 clades.

### L2.3.4 to L2.3.6 strains drive the global expansion of Beijing family strains.

**FIG 3:**
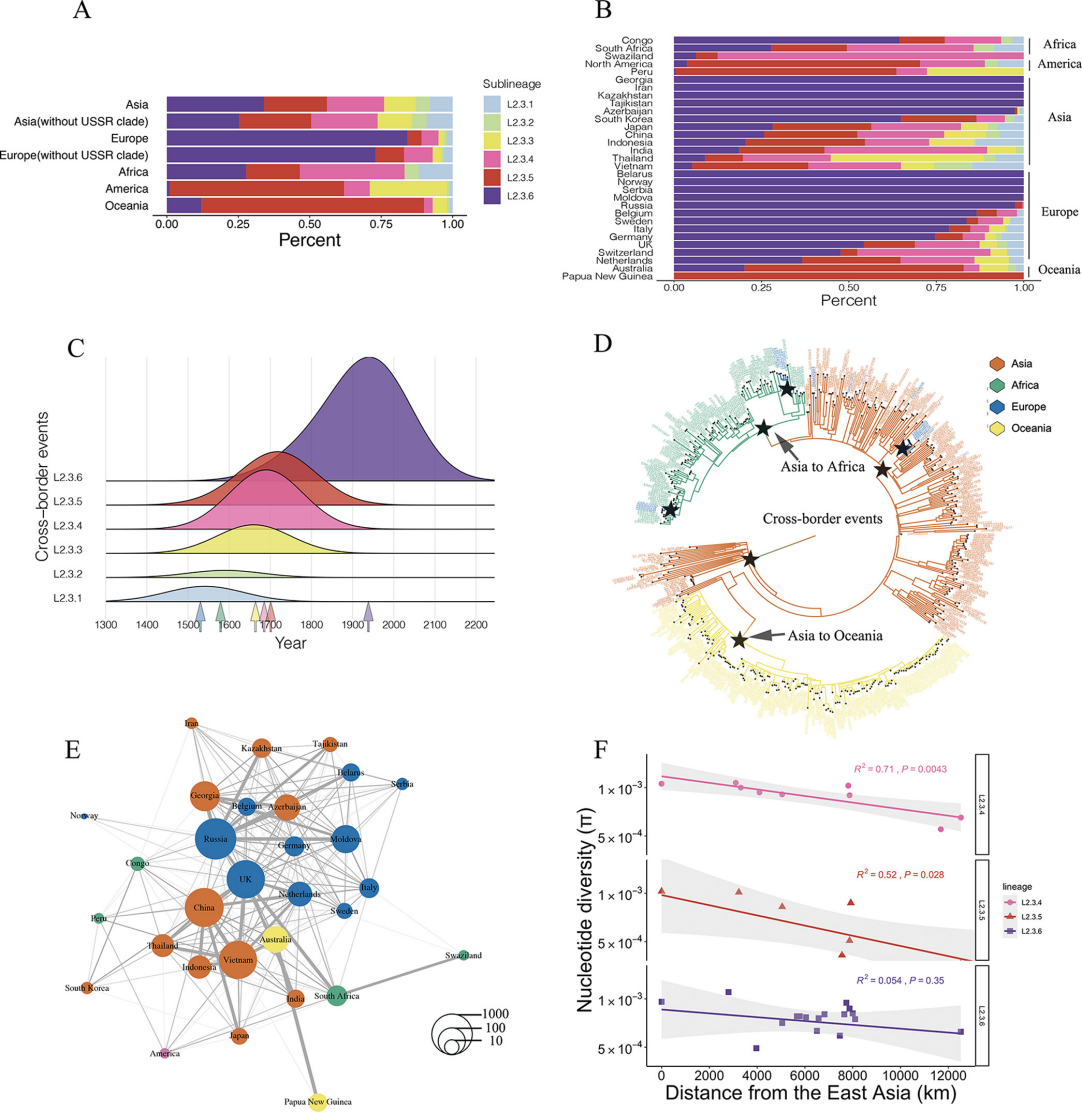
L2.3.4 to L2.3.6 clades drive the global expansion of Beijing family strains. (A) The relative proportion of L2.3.1 to L2.3.6 clades in six continents. (B) The relative proportion of L2.3.1 to L2.3.6 clades in different countries (only showed countries with sample size >30). (C) Inferred event density of cross-border transmission of the six L2.3 clades through time. (D) A representative phylogenetic tree indicating how the cross-border events were identified. (E) Inferred frequency of cross-border transmission events between countries. (F) Genetic erosion out of East Asia within clades L2.3.4 to L2.3.6.

Of all the L2.3 strains collected for this study, about 84.8% (6,693 of 7,896) belonged to the L2.3.4 to L2.3.6 clades, which were isolated from all 51 countries. In contrast, strains belonging to the L2.3.1 to L2.3.3 clades accounted for only 15.2% (1,203 of 7,896) of the collection and were found in just 28 of the 51 countries. To eliminate possible bias from L.2.3 strains such as W148 and central Asian (CAO) that caused large outbreaks after the collapse of the Soviet Union, we eliminated these strains from the analysis but again found that the percentage of L2.3.4 to L2.3.6 clades was consistently higher than the percentage of the other L2.3 clades. (Fig. 3A and B). Of all L2 strains isolated outside East Asia, 83.1% belonged to clades L2.3.4 to L2.3.6.

The broad global prevalence of clades L2.3.4 to L2.3.6 suggested that they could have a higher potential for cross-border transmission. We used BEAST to trace the timing of spread across countries and defined “cross-border events” based on the multicountry origin of MTBC strains on branches of the phylogenetic tree (Fig. 3C and D). By counting the time and frequency of the cross-border events of the different L2.3 clades, we found that the cross-border spread of clades L2.3.1 to L2.3.3 occurred relatively early after the clades diverged (1500 to 1650 AD). In contrast, the cross-border events of both L2.3.4 to L2.3.6 were more recent (1700 to 2000 AD) and more numerous (687 versus 148 events) (Fig. S4). While China had frequent cross-border strain exchanges with Southeast Asia, transmission to Europe and Central Asia was more frequent from Russia. The United Kingdom appeared to be a hub of cross-border transmission, presumably because it contained a variety of strains brought by infected immigrants from different geographic regions (28) (Fig. 3E; Fig. S5). Additionally, we investigated the relationship between the change in genetic diversity within the clades L2.3.4 to L2.3.6 and the distance from East Asia. We found that the genetic diversity decreased as the geographic distance increased, with more than 50% of the variance explained by geographic factors alone (Fig. 3F). This trend remained consistent even when we expand our sample size to encompass more countries (Fig. S6). For clade L2.3.6, however, the genetic diversity did not significantly change with geographic distance, perhaps due to the recent outbreak expansion of strains such as W148/CAO (shown in Fig. 2A). This suggests that the expansion center of L2.3.6 might be situated in Eastern Europe, consistent with the results of cross-border events (Fig. 3E; Fig. S5).

### Evolution of clades L2.3.4 to L2.3.6.

As clades L2.3.4 to L2.3.6 share an ancestor and diverged in parallel with clades L2.3.1 to L2.3.3 (Fig. 1B), two hypothetical scenarios might explain the evolution of their increased transmissibility: (i) founder effect, whereby the common ancestor of clades L2.3.4 to L2.3.6 acquired adaptive mutations so that the three clades share the same transmission advantage; or (ii) parallel evolution, whereby the three clades evolved increased transmissibility in parallel but presumably through different mutational paths.

**FIG 4:**
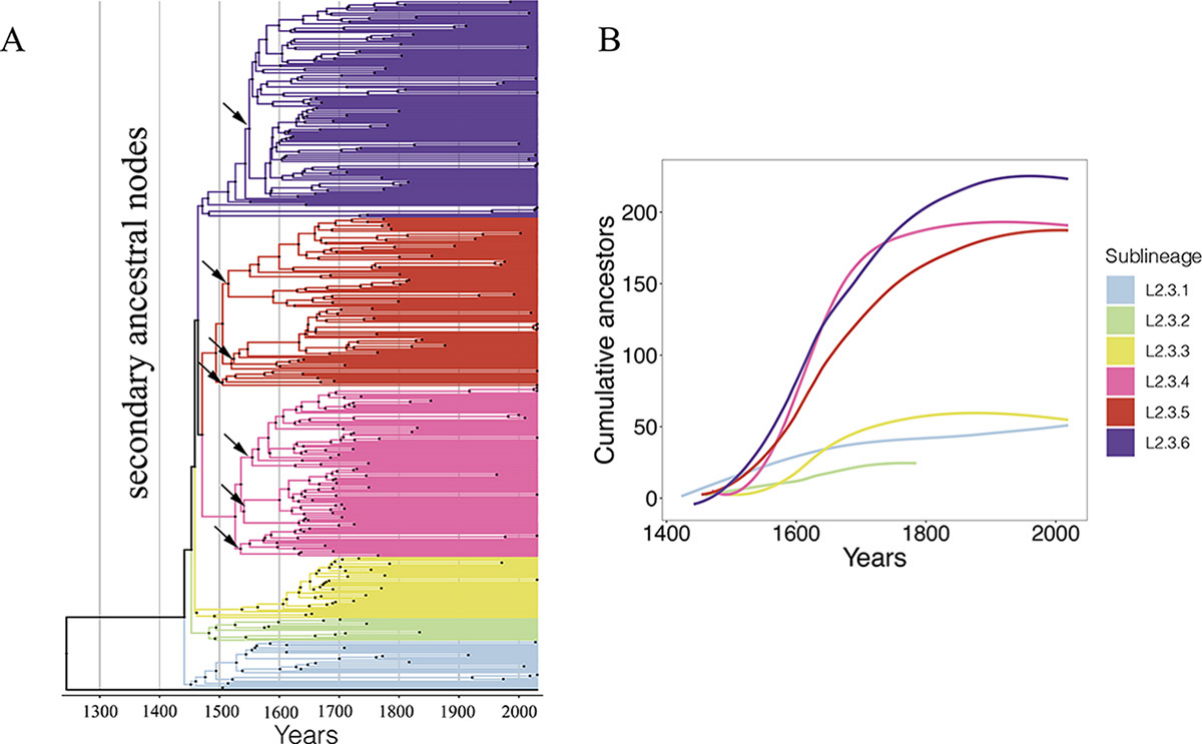
Accumulation of secondary ancestral nodes. (A) A Bayesian-estimated time-calibrated phylogenetic tree of L2.3 clades sampled from East Asia, with secondary ancestor nodes with comparable transmission advantage annotated. (B) Cumulative numbers of secondary ancestral nodes in the six L2.3 clades.

In the founder scenario, the expansion should have begun with the L2.3.4 to L2.3.6 common ancestor, and the secondary ancestral nodes should have similar expansion dynamics (Fig. 4A and B). Consistent with a founder effect, clades L2.3.4 and L2.3.5 have secondary ancestral nodes with similar patterns of expansion dynamics, and we found no differences in the geographic distribution or population sizes for the descendants of L2.3.4 to L2.3.5 (Fig. 2A). Clade L2.3.6, however, has distinct secondary ancestral nodes with a clear expansion only in the most recently diverged node and a geographically restricted distribution (Fig. 4A). These results suggest that the transmission advantage of L2.3.4 and L2.3.5 was likely acquired by their common ancestor, while the success of L2.3.6 may be the result of mutations acquired only at the latest evolved node (Fig. 4B).

We searched for the genetic variations that define each clade by reconstructing their ancestral sequences. We found that just 43 SNPs differentiated clades L2.3.1 to L2.3.6 from the last common ancestor, with 4, 5, 17, 13, 3, and 1 private SNPs, respectively (Table 2; Table S4), but the latest evolved node of L2.3.6 had 12 private SNPs (Table S5). Analysis of the function of the genes in which these SNPs occur suggested a selection for mutations in transmembrane-related pathways (Fig. S7). Finally, we investigated whether the mutated genes could be targets for positive selection by checking for genes that are known to have been subject to positive selection in the MTBC population (29) and found that *ftsK*, *fadE17*, and *Rv2209* showed positive selection, while the rest of the genes with SNPs in the L2.3 clades are under negative selection.

**TABLE 2:** Clade-specific mutations for L2.3.4 to L2.3.6

Clades	Gene	Position	Allele change	Codon change	Gene description
L2.3.4	*trpG*	15087	C/G	T58T	Possible anthranilate synthase component II TrpG (glutamine amidotransferase)
	*mpa*	868663	G/A	S52S	Mycobacterial proteasome ATPase Mpa
	*Rv1231c*	1279968	G/C	L60L	Probable membrane protein
	*kshB*	1374685	C/G	L198L	Reductase component of 3-ketosteroid-9-α-hydroxylase KshB
	*mscR*	1451405	G/A	E124E	*S*-Nitrosomycothiol reductase MscR
	*Rv2910c*	2185884	T/C	D122D	Conserved hypothetical protein
	*ftsK*	2376135	A/G	A268A	Possible cell division transmembrane protein FtsK
	*Rv3730c*	2532616	G/A	A294A	Conserved hypothetical protein
	*thrC*	3061703	G/A	G237S	Threonine synthase ThrC (ts)
	*echA21*	3217905	G/A	G124D	Possible enoyl-CoA hydratase EchA21 (enoyl hydrase) (unsaturated acyl-CoA hydratase) (crotonase)
	*Rv1152*	4013010	G/A	G105A	Probable transcriptional regulatory protein
	*fadE17*	4180839	G/C	N102S	Probable acyl-CoA dehydrogenase FadE17
	*Rv0775*	4219219	G/A	R86Q	Conserved hypothetical protein
L2.3.5	*Rv2209*	2474686	C/T	A429A	Probable conserved integral membrane protein
	*serA1*	3355057	C/T	V5I	Probable d-3-phosphoglycerate dehydrogenase SerA1 (PGDH)
	*Rv3770c*-*Rv3770A*	4215793	A/C		*Rv3770c*: Hypothetical leucine-rich protein
					*Rv3770c*: Probable remnant of a transposase
L2.3.6	*Rv3603c*	4046007	C/T	V38M	Conserved hypothetical alanine- and leucine-rich protein

## DISCUSSION

In this study, we used phylogenetic branching and population dynamics to demonstrate that L2.3 (modern Beijing) diversified into six phylogenetic clades associated with various transmission characteristics. MTBC strains belonging to the L2.3.4 to L2.3.6 clades had higher clustering rates in the population-based data sets than strains belonging to clades L2.3.1 to L.2.3.3, indicating higher levels of transmission. In addition, compared to the other L2.3 clades, L2.3.4 to L2.3.6 are more widely distributed globally and were associated with more cross-border transmission events. These results suggest that the global success of L2.3 was not caused by the entire sublineage but rather was due to the rapid expansion of just three clades: L2.3.4 to L2.3.6.

The MTBC strains of the Beijing family have spread throughout the globe, causing both endemic disease and outbreaks with highly transmitted, often drug-resistant strains (30–32). The Beijing genotype was first identified by finding highly conserved spoligotypes and IS6110 RFLP patterns in MTBC isolates from Mongolia and the Beijing region of China (9). Subsequently, the Beijing family was divided into the ancient Beijing (later termed L2.2) and modern Beijing (L2.3) sublineages based on the absence or presence of an IS6110 insertion in the NTF region (18, 33, 34). We have used the term “L2.3” rather than “modern Beijing,” because although L2 was originally found in strains from the Beijing region, L2 strains, especially those belonging to the L2.3 sublineage, are found in many countries. In addition, we aim to use a systematic nomenclature to avoid discrimination against specific cities (35).

L2.3 strains have been shown to be more virulent than the L2.2 strains in both mouse and macrophage infection models (20, 36). Some of the increased virulence of L2 strains might be explained by their tendency to accumulate more triacylglycerol (TAG) than other lineages, which could facilitate their growth within the host and promote rapid disease progression (37). Other possible markers of virulence include the ability of L2.3 strains to grow faster *in vitro* and trigger a vigorous immune response with pronounced macrophage infiltration (38). However, previous studies comparing the phenotypic differences between L2.2 and L2.3 strains have regarded the L2.3 sublineage as a homogeneous group, but our findings suggest that the different clades of L2.3 do not all share the same pathogenic and transmission characteristics. We suggest that future studies to elucidate the pathogenicity of the L2.3 sublineage should concentrate on strains belonging to the more successful L2.3.4 to L2.3.6 clades.

It was recently proposed that factors such as sampling bias and molecular clock rate may bias estimations of recent transmission based on clustering rate or terminal branch length (39). However, our clustering rates are based on population-based studies with an unbiased sampling of the different L2.3 clades, and we used different clustering thresholds to assess recent transmission rates. There is still disagreement on the correct molecular clock rate for tracing MTBC evolution, but we used historical population expansions to demonstrate differences between the L2.3 clades. Therefore, our findings should be robust to various molecular clock rates that may change the time scale but not the relative differences between L2.3 clades.

Drug resistance is another source of potential bias, as rapid resistance development and compensatory evolution can play a significant role in bacterial dissemination (40). However, although drug resistance could have promoted recent expansions, the Bayesian-based analysis showed that the main, historic population expansions of L2.3.4 to L2.3.6 occurred before 1800, well before the advent of antibiotics. Other epidemiological and social factors may also contribute to the spread of the bacteria (41). Within L2.3.6, there are two subclades with shorter branch lengths: one with strains from W148 and the other with strains from CAO (25) (Fig. S8). The expansions of W148 and CAO were associated with inadequate health services after the collapse of the former Soviet Union (42). However, although the W148 and CAO subclades together accounted for 36.5% of the global L2.3.6 population, they do not completely explain the success of the L2.3.6 clade, as other subclades had already spread widely outside Eurasia.

Interestingly, although there have been outbreaks with strains from both the L2.3.4 and L2.3.5 clades, the diversity of the strains in these clades progressively decreased as the geographic distance from East Asia increased. In contrast, L2.3.6 strains maintained their diversity even as the distance from East Asia increased (Fig. S6). This suggests that the expansion center of L2.3.6 might be situated in Eastern Europe, with Russia serving as the focal point for the cross-border transmission of L2.3.6. While social and epidemiological factors may have played a role in some local population expansions, there were many more L2.3.4 to L2.3.6 strains than L2.3.1 to L2.3.3 strains in every one of the nine countries where all six clades were present. This implies that differences in the success of the six clades are due to as-yet-unidentified genetic determinants of transmission rather than host, geographic, or epidemiologic factors. Understanding the molecular mechanisms responsible for the L2.3.4 to L2.3.6 clades might help trace the evolution of MTBC pathogenicity.

Over the course of the severe acute respiratory syndrome coronavirus-2 (SARS-CoV-2) pandemic (43, 44), the ability to monitor the evolution of this pathogen in real time has shown that a few genetic variations can modify the characteristics of its transmission and pathogenicity. Although the evolution of MTBC occurred over hundreds or thousands of years, insights provided by phylogenetic reconstruction and estimation of *N_e_* dynamics suggest that MTBC populations were similarly shaped by the evolutionary acquisition of mutations. Examination of the private SNPs accumulated by clades L2.3.4 to L2.3.6 suggests a positive selection for mutations in transmembrane-related pathways, which might be interesting to explore in future studies.

In conclusion, we found that the L2.3 sublineage can be divided into six distinct clades, and the global spread of the Beijing Family was mostly due to the transmission success of just the three most recently emerged clades. Highly transmissible MTBC strains play a large role in the global TB epidemic, and therefore understanding the functional and molecular basis of transmission could help formulate targeted TB prevention and control strategies.

## MATERIALS AND METHODS

### Collection of genome sequences of MTBC L2.3 isolates.

We searched PubMed for articles with whole-genome sequencing data published from 2005 to 2020 (Table S6) and then downloaded the original sequencing reads, as described in a recent study by Liu et al. (45). The following two SNP-typing schemes were implemented to identify the strains in the resulting collection: the phylogenetically informative SNPs derived by Coll et al. (46) from a global collection of L2 strains and 44 SNPs specific to modern Beijing strains (L2.3) described by Liu et al. (19). The collection used for the study included WGS data of 7,896 MTBC L2.3 strains isolated from a total of 51 countries in Asia, Europe, Oceania, Africa, and the Americas (Table S1). The collection included strains isolated in large outbreaks, some involving highly clonal strains. The sequencing data were generated on the Illumina platform and derived from over 70 studies.

### Variant calling.

We used a previously validated pipeline for mapping short sequencing reads to the reference genome (8). The Sickle (47) tool was used to trim the WGS data and sequencing reads with a Phred base quality above 20, and read lengths longer than 30 were kept for analysis. Bowtie 2 (version 2.2.9) (48) was used to map sequencing reads to M. tuberculosis H37Rv strain (NC_000962.2) as a reference template. SAMtools (version 1.3.1) (49) was used for SNP calling with mapping quality greater than 30, and fixed mutations (frequency ≥75%) were identified using VarScan (version 2.3.9) (50). We discarded low-quality isolates with an average sequencing depth lower than 20 and excluded SNPs in repetitive regions of the genome (PPE/PE-PGRS family genes, phage sequences, insertions, or mobile genetic elements) and identified small indels.

### Phylogenetic reconstruction.

To perform phylogenetic reconstruction, the SNPs of the MTBC isolates were combined into a single consensus and nonredundant list, while nucleotide positions with gaps in more than 5% of the taxa were excluded as possibly due to insertions or deletions, low coverage, or poor mapping quality at those sites. A maximum-likelihood (ML) phylogenetic tree with alignments of the 7,896 isolates was inferred using RAxML v.8.2.12 (51) with the general time-reversible (GTR) model of nucleotide substitution and γ rate heterogeneity with four γ classes. The neighbor-joining method was used for the initial inference of the phylogenetic structure for large numbers of taxa. To test the bootstrap, a ML phylogenetic tree of 7,896 isolates worldwide was inferred using IQ-TREE version 2 (52) with ultrafast bootstrap supports from 1,000 replications. The best-fit nucleotide substitution model was GTR+I+G, as determined by ModelFinder (53) (Fig. S9). Phylogeny trees were visualized in FigTree (version 1.4.3) or iTOL (54).

### Definition of phylogenetic clades of L2.3.

We defined L2.3 clades based on the following criteria: (i) isolates forming a monophyletic clade in the phylogenetic tree; (ii) clades with bootstrap support for at least 90% at the branch; and (iii) all isolates within a clade share at least one common SNP not present in the other clades.

### Phylogeographic analysis.

RASP (26) was used to estimate the ancestral geographic extent of MTBC L2.3. To estimate the geographic origins of each L2.3 clade, we divided the world map into five broad geographic areas and used them as proxies for the most likely origin of each strain. RASP reconstructions were performed by loading the maximum-likelihood phylogeny of the lineages and the corresponding geographic regions of origin of the isolates as distributions. For the Bayesian-based analyses, three different chains were run for 30,000,000 generations. After three random sampling tests, we found that the geographical branches located at the end of the phylogenetic tree had almost no effect on the inference of the origin of the roots. Because there were more East Asian strains than the sample size limitation (<500 cases) of the RASP software, RASP was run with 485 representative East Asian strains that are closer to the root in the phylogenetic structure and did not belong to outbreaks (Fig. S1).

### Pairwise SNP distance.

A Perl script was written to calculate the SNP difference between all possible strain pairs and perform cluster analysis according to thresholds of 6 and 12 SNPs. We selected 29 countries with more than 30 isolates in the collection (Table S1) and calculated the pairwise SNP distances grouped by different clades. The distribution and mean pairwise SNP distance for each country were plotted with ggplot2 in RStudio (version 3.4.0) (55), and a Wilcoxon rank-sum test was used to evaluate the differences between countries.

### Ancestral reconstruction.

The ancestral sequences were reconstructed for clades L2.3.1 to L2.3.6 using PAML software (56). The Baseml method was used to analyze the alignment fasta file and generate an ML tree with the general time-reversible model of nucleotide substitution and γ distribution to account for rate heterogeneity between sites. A Perl script was written to identify the SNPs that were uniformly presented in all L2.3 strains but absent in the closest L2.2 strains. Support values for each site were >90% for all analyses.

### Terminal branch lengths.

Terminal branch lengths are reported as the number of substitutions (SNPs) mapped to each terminal branch. Briefly, to estimate the terminal branch lengths, the alignment lengths of variable positions for each strain were used as surrogates. We wrote a script to count the number of SNPs accumulated by each strain after differentiation from the common ancestor as branch lengths (SNPs) (data in Fig. 2B). The terminal branch lengths between the different clades were compared using a Kolmogorov-Smirnov test in Python (version 3.7.0). Clusters were defined as the number of strain pairs on the descendant tips of the tree with SNP distances below a specified threshold. We calculated the cluster sizes for each clade at successive thresholds increasing by five SNPs (data in Fig. 2C). Studies focusing on large outbreaks or highly clonal strains were not included.

### Biogeography.

We utilized Mega X software (57) to evaluate the diversity (π) of each of the samples collected from various geographic regions. We analyzed 9 countries for L2.3.4, 9 countries for L2.3.5, and 18 countries for L2.3.6, including only those countries with a sample size greater than 30 (refer to Table S8 and S9 for details). In addition, we resampled countries with a sample size greater than 50. For countries with sample sizes between 30 and 50, we included all isolates in the analysis. We then calculated the geographic distance between each location and northern East Asia, near Beijing, using the shortest walking distance based on classic human migration routes, as determined by tools available on Google Earth. Finally, we computed the linear correlation (*r*^2^) between the diversity (π) of each area and its geographical distance from the source.

### Dating analysis.

We estimated the dates of the most common recent ancestors of L2.3 and their sublineages using BEAST (version 1.8.0) (58). The XML input file was modified to specify the number of invariant sites in the MTBC genomes. For the MTBC genome substitution rate, we imposed a normal distribution for the substitution rate of MTBC with a mean of 4.6 × 10^−8^ substitutions per genome per site per year, as used in a previous study (59). A strict distribution was used for the substitution rate with a constant population size for the tree priors. We ran 3 chains of 30,000,000 generations to ensure independent convergence of the chains, the first 10% of which were discarded as burn-in. Convergence was assessed using Tracer (version 1.6.0) to ensure that all relevant parameters reached effective sample size (ESS) values greater than 200. Phylogenetic trees were visualized using iTOL (54).

### Migration rate inference.

Migration rates over time were inferred from the Bayesian maximum clade credibility tree (MCC) using the results from the analyses above. Only nodes with posterior probabilities greater than or equal to 80% were considered. A migration event was defined as a change from the most probable reconstructed ancestral geographic region of a parent strain to a different geographic region for strains in the descendant node. We identified migration events as a change in the most probable reconstructed ancestral geographic region from a parent to a child node in the phylogenetic tree (60). The median heights of the parent and descendant nodes were treated as a range of time over which the migration event could have occurred (Table S7). The rate of migration over time for each clade was inferred by summing the number of migration events occurring across each year of the time-scaled phylogeny (60). The results of these analyses were visualized using TABLEAU (61) and R software (55).

### Bayesian skyline plot.

Bayesian skyline plot (BSP) analysis was applied to estimate the effective past population size dynamics of the six L2.3 clades based on the substitution rate model indicated above. Skyline analysis was performed separately for all East Asian L2.3 strains based on clades L2.3.1 to L2.3.6, with the age of their most recent common ancestor used as the tree height. In each case, 3 chains of 30,000,000 generations were sampled every 1,000 generations to ensure independent convergence of the chains. For the population dynamics plot in Fig. 1C, the relative prevalence of each clade in the past was estimated individually from the effective population growth curves generated by BSP analysis and plotted using the streamgraph package in RStudio (version 3.4.0) (55).

### Population growth rate estimation.

The population growth rate per year was calculated using the effective population growth curves generated from the BSP analysis (62). Each skyline plot consisted of 100 smoothed data points at 10-year intervals. The initial population size *N*_0_ was set as the time when the different clades emerged, and we estimated the effective population growth rate for the increasing interval in our data using the exponential growth equation:

$$R_{\mathrm{Ne}}=\;\ln\;\left[N_{t}/N_{0}\right]/t$$

In this equation, *R_Ne_* represents the population growth rate per year, *N* is the initial population size, and *t* is the duration of time since growth began.

### Gene enrichment analysis.

Gene Ontology (GO) and KEGG analyses were performed on the locations of the private mutations of each clade, L2.3.1 to L2.3.6, using the online software David (https://david.ncifcrf.gov/summary.jsp). The private mutations of each of the six clades were assembled into a list and uploaded to the David software with the MTBC H37Rv strain as a reference. The gene ratio and rich factors for each clade were visualized with ggplot2 in RStudio (version 3.4.0) (55).

### Data availability.

The raw data including (i) the fasta file containing the aligned and concatenated SNP sequences used to construct the phylogenetic trees presented in Fig. 1B and 2; (ii) the XML file for the Bayesian skyline plot analysis; and (iii) the original tree files generated by IQ-Tree, with bootstrap values indicated have been uploaded to figshare (https://figshare.com/projects/The_global_success_of_Mycobacterium_tuberculosis_Modern_Beijing_family_is_driven_by_a_few_recently_emerged_strains/162727).
