# Supplementary material for: The Global Success of Mycobacterium tuberculosis Modern Beijing Family Is Driven by a Few Recently Emerged Strains
Source: Microbiol Spectr. 2023 Jun 5;11(4):e03339-22. doi: 10.1128/spectrum.03339-22 (PMC10434187; doi:10.1128/spectrum.03339-22)
Supplement: Supplemental file 1 — Supplemental material. Download spectrum.03339-22-s0001.pdf, PDF file, 3.4 MB [file spectrum.03339-22-s0001.pdf]

Supplementary Table 1 Global L2.3 Strains Collection

Continent	Count	Percent
Africa	652	8.3%
America	258	3.3%
Asia	3805	48.2%
Europe	2945	37.3%
Oceania	236	2.9%
All	7896	100%

Supplementary Table 2 The proportion of global prevalent L2.3 within L2

Country (East Asia/ Southeast Asia)	Percent	Country (Central Asia)	Percent	Country (Outside Asia)	Percent
China	75.0%	Nepal	93.0%	Russia	93.8%
Japan	57.5%	Pakistan	100.0%	Belarus	100.0%
South Korea	54.7%	Afghanistan	100.0%	Australia	76.2%
Vietnam	78.5%	Iran	96.7%	Papua New Guinea	96.9%
Thailand	69.8%	Turkmenistan	81.8%	Uganda	100.0%
Myanmar	82.8%	Uzbekistan	100.0%	Malawi	100.0%
Philippines	75.0%	Kazakhstan	98.4%	Kenya	100.0%
Indonesia	69.3%	Azerbaijan	100.0%	Mozambique	100.0%
India	84.7%	Georgia	73.3%	South Africa	76.8%
				Swaziland	100.0%

Supplementary Table 3 Comparison of clustering rates of different sublineages in China cohort

Locate\Cutoff		6-snp threshold			12-snp threshold		
		Cluster	non-Cluster	*P Value*	Cluster	non-Cluster	*P Value*
Baoan	L2.3.1-L2.3.2	3	108	ref	7	94	ref
	L2.3.4-L2.3.6	73	657	0.0235	126	604	0.0121
	L4.2	6	66	0.1577	8	64	0.4908
	L4.4	14	171	0.1379	20	165	0.3892
	L4.5	19	163	0.0271	20	162	0.3670
	L2.2	44	338	0.1634	50	332	0.1254

Supplementary Table 4 Clade-specific mutations for L2.3.1-L2.3.3

Clades	Gene	Position	Allele change	Codon change	Gene description
L2.3.1	gpdA1	655559	G/A	P131S	Probable glycerol-3-phosphate dehydrogenase [NAD(P)+] GpdA1 (NAD(P)H-dependent glycerol-3-phosphate dehydrogenase) (NAD(P)H-dependent dihydroxyacetone-phosphate reductase)
	gnd1	2093991	C/T	A400T	Probable 6-phosphogluconate dehydrogenase Gnd1
	dacB2	3218997	T/A	L220Q	Probable penicillin-binding protein DacB2 (D-alanyl-D-alanine carboxypeptidase) (DD-peptidase) (DD-carboxypeptidase) (PBP) (DD-transpeptidase) (serine-type D-ala-D-ala carboxypeptidase) (D-amino acid hydrolase)
	atsB	3684649	G/A	R439W	Probable arylsulfatase AtsB (aryl-sulfate sulphohydrolase) (sulfatase)
L2.3.2	bioF2	36577	C/T	A761A	Possible 8-amino-7-oxononanoate synthase BioF2 (AONS) (8-amino-7-ketopelargonate synthase) (7-keto-8-amino-pelargonic acid synthetase) (7-KAP synthetase) (L-alanine--pimelyl CoA ligase)
	inhA	1674210	A/C	G3G	NADH-dependent enoyl-[acyl-carrier-protein] reductase InhA (NADH-dependent enoyl-ACP reductase)
	Rv1565c	1772288	T/G	A514A	Conserved hypothetical membrane protein
	Rv3160c	3529598	C/G	E128Q	Possible transcriptional regulatory protein (probably TetR-family)

	Rv3401	3818073	C/A	P11Q	Conserved protein
L2.3.3	cmaA2	284588	C/T	S143S	Cyclopropane-fatty-acyl-phospholipid synthase 2 CmaA2 (cyclopropane fatty acid synthase) (CFA synthase) (cyclopropane mycolic acid synthase 2) (mycolic acid trans-cyclopropane synthetase)
	pstC2	594351	T/C	L186L	Phosphate-transport integral membrane ABC transporter PstC2
	coaA	787318	G/T	G168G	Probable pantothenate kinase CoaA (pantothenic acid kinase)
	Rv3728	829698	C/A	G403G	Probable conserved two-domain membrane protein
	fadE31	1036583	C/T	N153N	Probable acyl-CoA dehydrogenase FadE31
	Rv2980	1219683	G/A	R134R	Possible conserved secreted protein
	serB2	1600039	G/T	A408A	Probable phosphoserine phosphatase SerB2 (PSP) (O-phosphoserine phosphohydrolase) (pspase)
	Rv0687	3300658	G/C	V74L	Probable short-chain type dehydrogenase/reductase
	Rv3689	3302485	G/A	Y410S	Probable conserved transmembrane protein
	Rv3479	3336359	C/A	F446L	Possible transmembrane protein
	aftD	3401939	G/A	G755E	Possible arabinofuranosyltransferase AftD
	Rv1425	3428183	G/A	G128C	Possible triacylglycerol synthase (diacylglycerol acyltransferase)
	Rv0737	3897155	T/C	D164E	Possible transcriptional regulatory protein
	cstA	4003619	C/T	R314H	Probable carbon starvation protein A homolog CstA
	fadD29	4131585	A/C	R600G	Fatty-acid-AMP ligase FadD29 (fatty-acid-AMP synthetase) (fatty-acid-AMP synthase)
	fadD29-Rv2951c	4176081	C/T	---	fadD29: Fatty-acid-AMP ligase FadD29 (fatty-acid-AMP synthetase) (fatty-acid-AMP synthase). Rv2951c: Possible oxidoreductase
	eccCb1-PE35	4350639	A/G	---	eccCb1: ESX conserved component EccCb1. ESX-1 type VII secretion system protein. PE35: PE family-related protein PE35

Supplementary Table 5 L2.3.6 Private mutation of secondary ancestral nodes

Gene	Position	Codon change	Allele change	Gene description
Rv0139	166565	I247V	A/G	Possible oxidoreductase
Rv0845	908186	I178S	T/C	Possible two-component sensor kinase
kdpD	941722	R128L	T/G	Probable sensor protein KdpD
argS	1151304	A119T	C/A	Probable arginyl-tRNA synthetase ArgS (ARGRS) (arginine--tRNA ligase)
Rv2522c	1341044	M215I	T/C	Conserved hypothetical protein
Rv2743c	1446733	P156T	G/A	Possible conserved transmembrane alanine-rich protein
Rv3770c	1722228	A115T	A/C	Hypothetical leucine-rich protein
Rv0260c	2838897	T257T	C/G	Possible transcriptional regulatory protein
guaA	2867207	V27V	C/G	Probable GMP synthase [glutamine-hydrolyzing] GuaA (glutamine amidotransferase) (GMP synthetase)
moeY	2881974	A72A	G/A	Possible molybdopterin biosynthesis protein MoeY
Rv2957	3056767	L235L	G/T	Possible glycosyl transferase
Rv2558-Rv2559c	3135950	+9-21-	---	Rv2558: Conserved protein Rv2559c: Conserved hypothetical alanine leucine valine rich protein

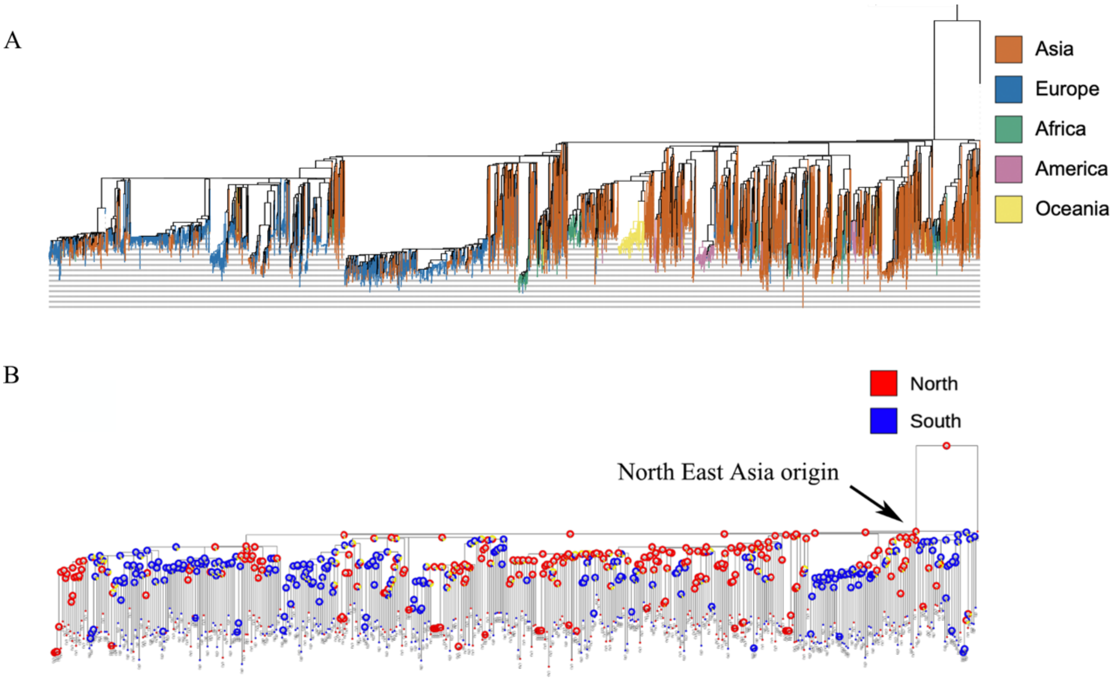

Figure S1 Global strain sampling A. phylogenetic tree with global strains B. RASP tree with East Asian strains

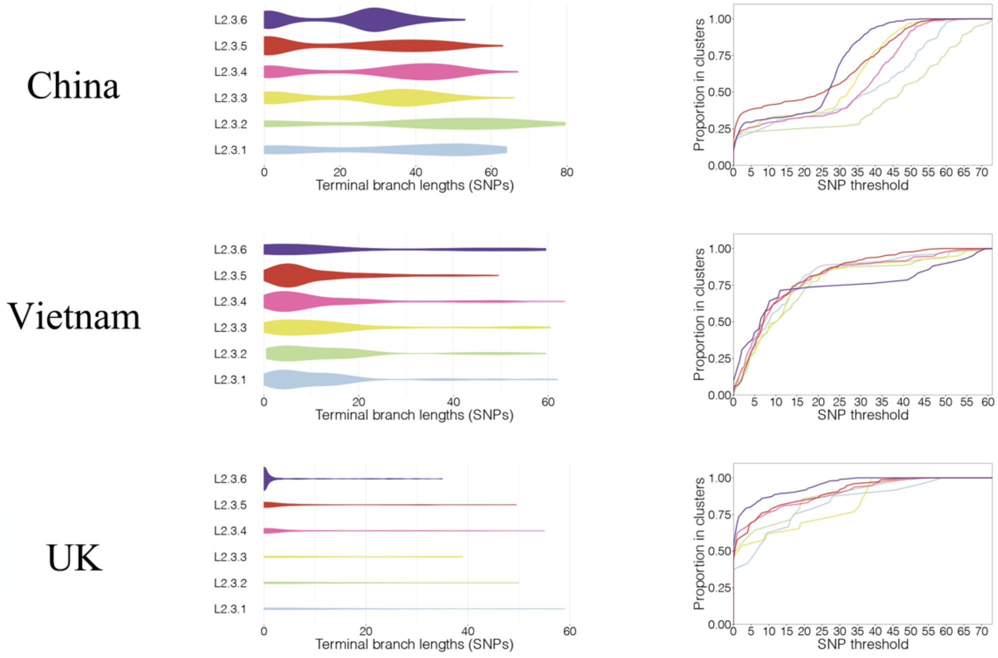

Figure S2 TBL and cluster size in different countries

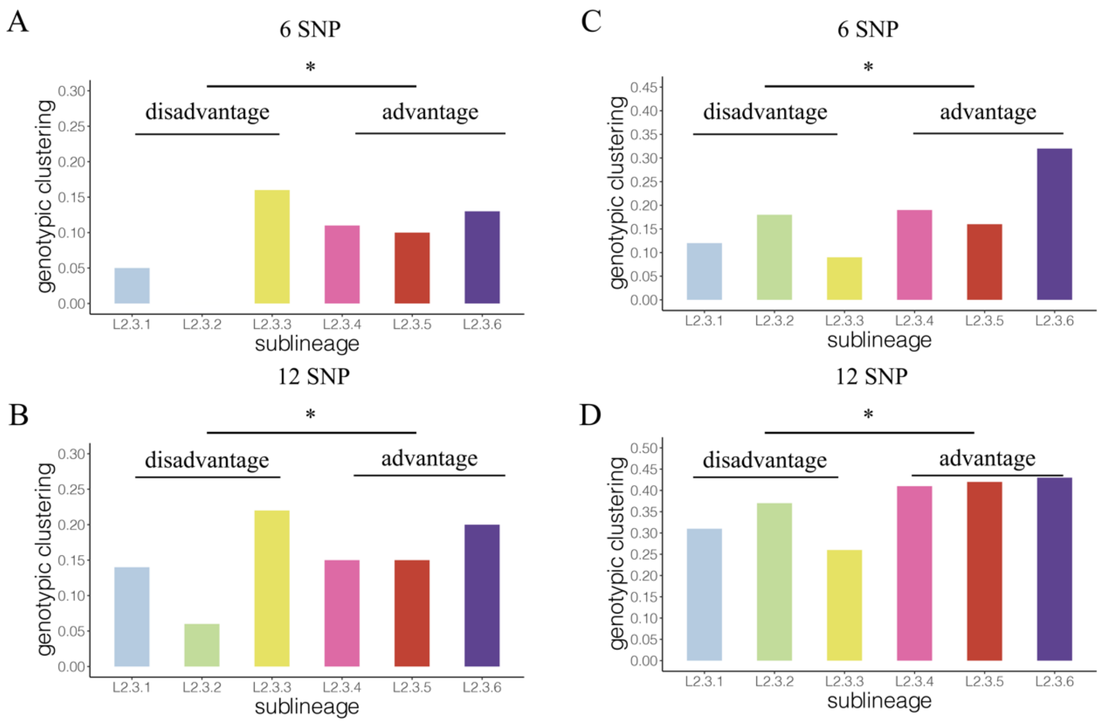

Figure S3 Other two clustering rates in China and Vietnam

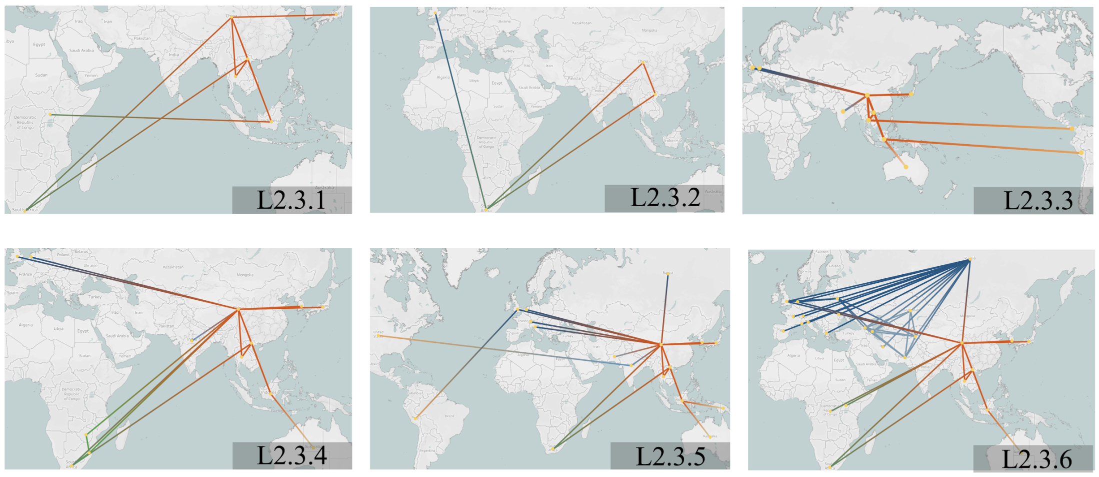

Figure S4 Geographical extent of global dissemination of six clades

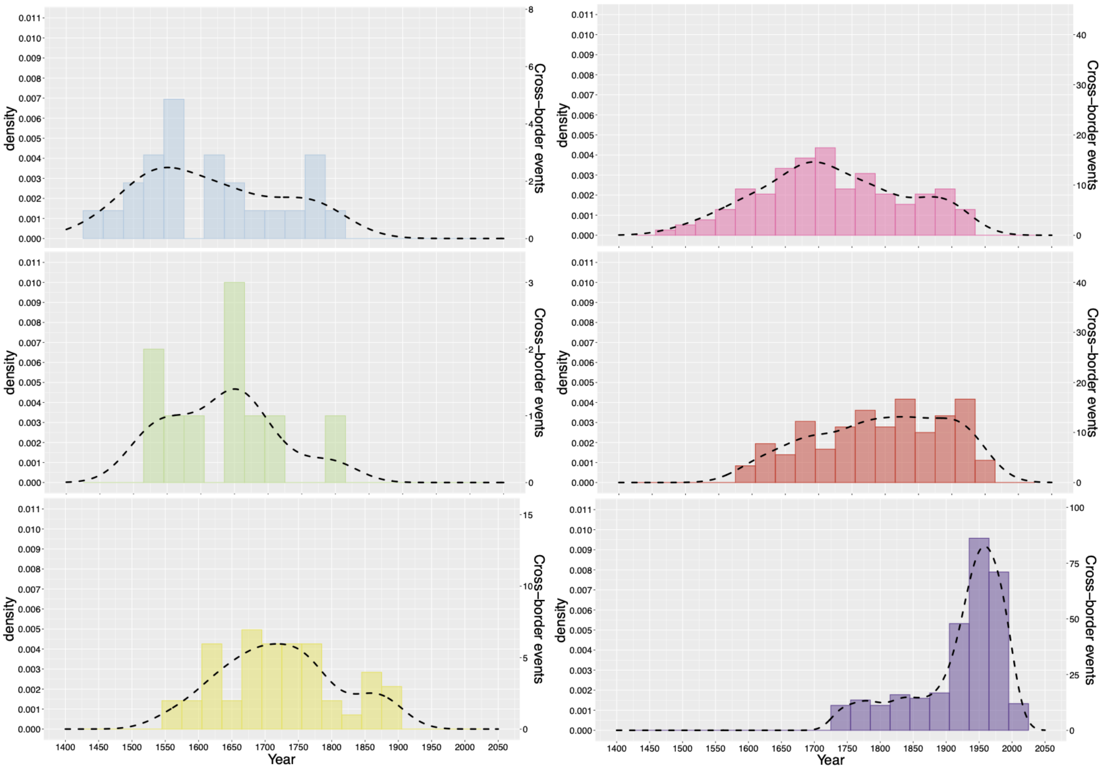

Figure S5 Cross-border events of six clades

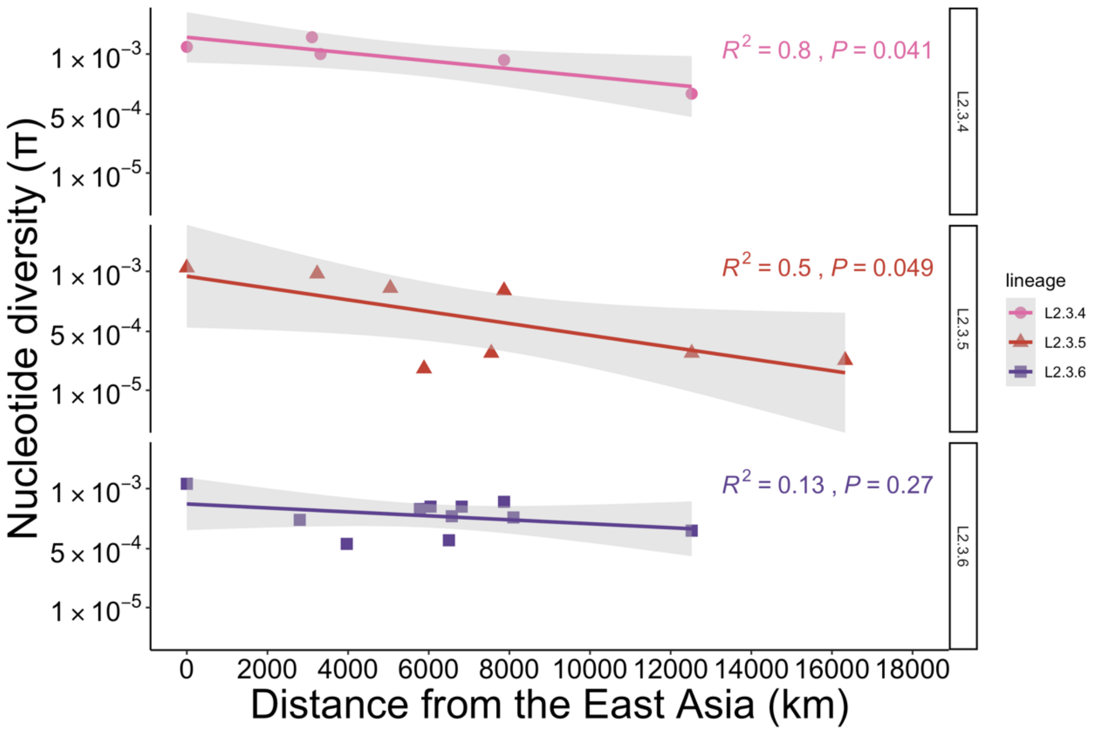

Figure S6 Genetic erosion out of East Asia within L2.3.4-L2.3.6. Countries with a sample size greater than 50.

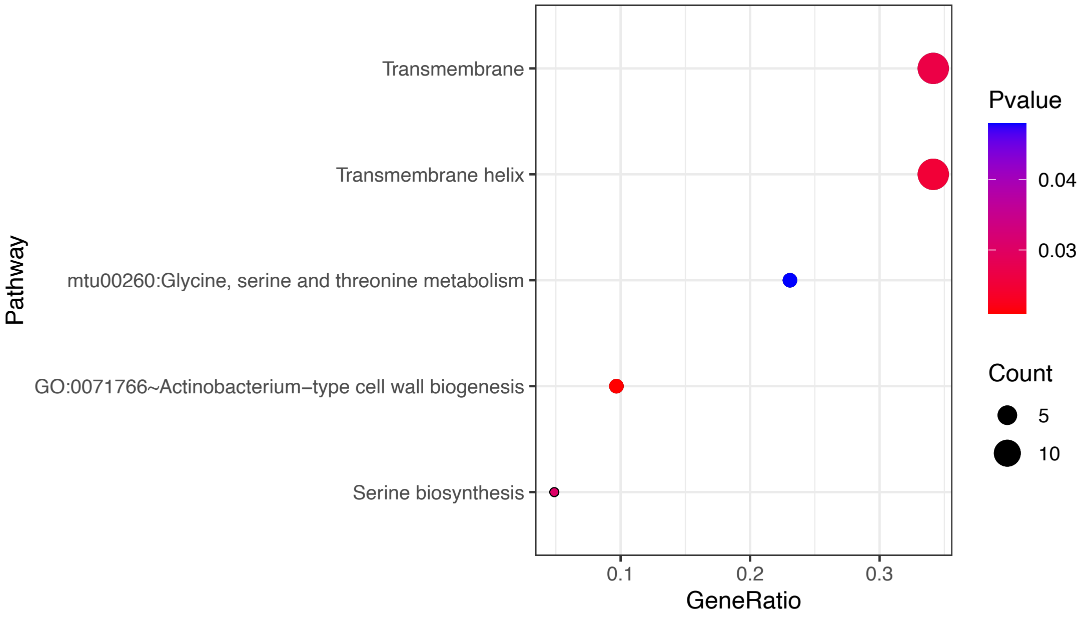

Figure S7 Clades L2.3.4-L2.3.6 gene enrichment

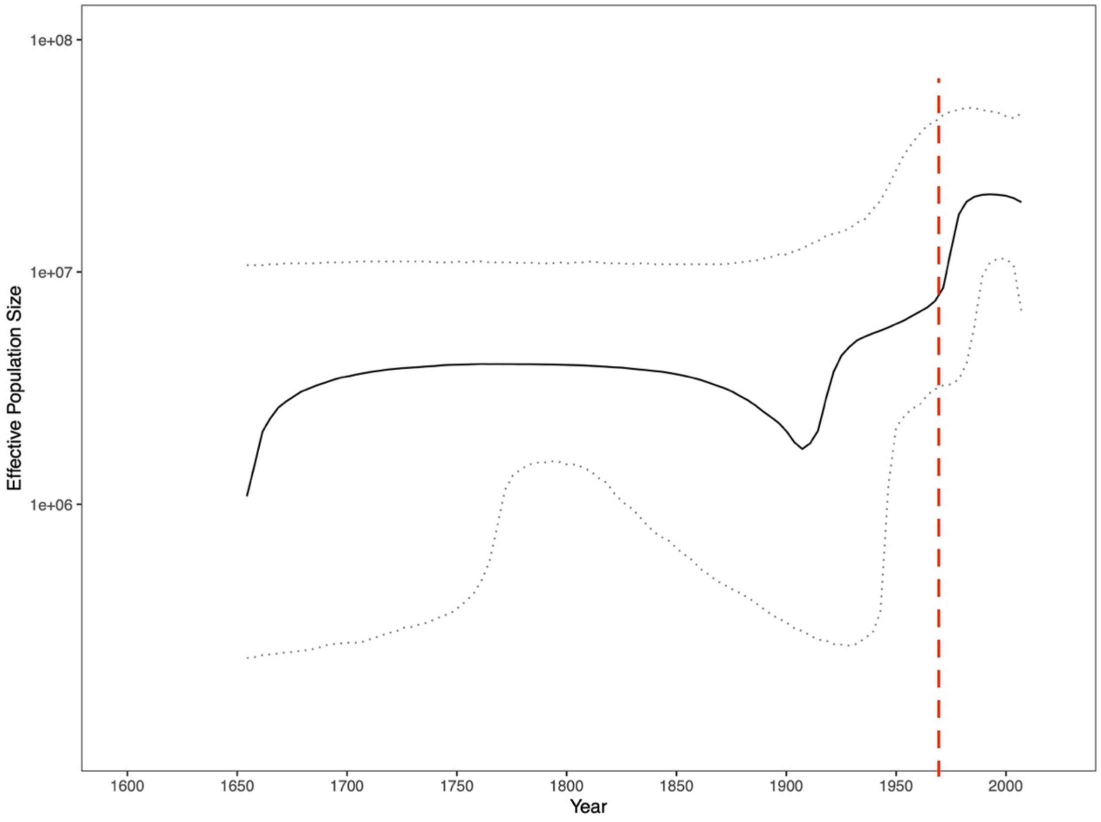

Figure S8 Bayesian skylines of three outbreak clades inside L2.3.6

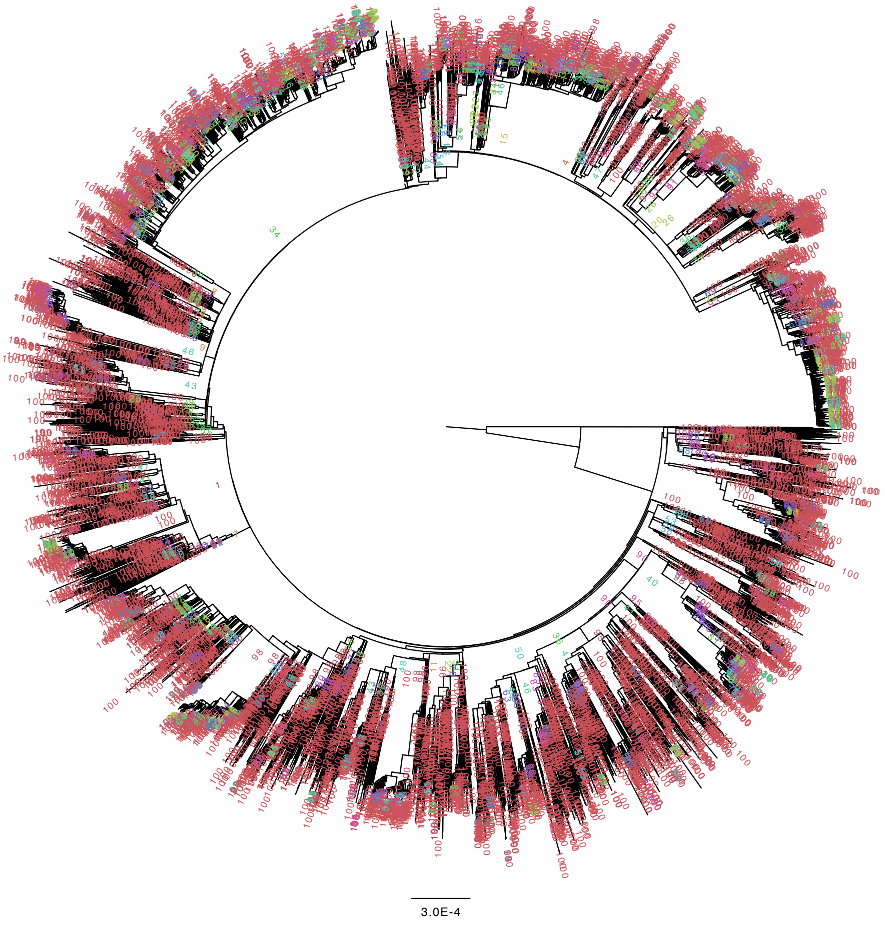

Figure S9 Phylogenetic tree with bootstrap
